# Contribution of Global and Regional Longitudinal Strain for Clinical Assessment of HFpEF in Coronary and Hypertensive Patients

**DOI:** 10.3390/medicina57121372

**Published:** 2021-12-17

**Authors:** Gheorghe Stoichescu-Hogea, Florina Nicoleta Buleu, Ruxandra Christodorescu, Raluca Sosdean, Anca Tudor, Andreea Ember, Daniel Miron Brie, Simona Drăgan

**Affiliations:** 1 Department of Cardiology, “Victor Babes” University of Medicine and Pharmacy, E. Murgu Square No. 2, 300041 Timisoara, Romania; goguhogea@yahoo.com (G.S.-H.); ralusosdean@yahoo.com (R.S.); pavellicuta@yahoo.com (A.E.); brie_daniel@yahoo.com (D.M.B.); simona.dragan@umft.ro (S.D.); 2 Department of Internal Medicine, “Victor Babes” University of Medicine and Pharmacy, E. Murgu Square No. 2, 300041 Timisoara, Romania; 3 Institute of Cardiovascular Diseases Timișoara, 13 Gheorghe Adam Street, 300310 Timișoara, Romania; 4 Department of Functional Sciences, “Victor Babes” University of Medicine and Pharmacy, E. Murgu Square No. 2, 300041 Timisoara, Romania; atudor@umft.ro

**Keywords:** myocardial strain, heart failure with preserved ejection fraction, hypertension, speckle-tracking echocardiography

## Abstract

*Background:* Contribution of global and regional longitudinal strain (GLS) for clinical assessment of patients with heart failure with preserved ejection fraction (HFpEF) is not well established. We sought to evaluate subclinical left ventricular dysfunction secondary to coronary artery disease (CAD) in HFpEF patients compared with hypertensive patients and age-matched healthy subjects. *Material and methods:* This was a retrospective study that included 148 patients (group 1 = 62 patients with HFpEF, group 2 = 46 hypertensive patients, and group 3 = 40 age-matched control subjects). Peak systolic segmental, regional (basal, mid, and apical), and global longitudinal strain were assessed for each study group using two-dimensional speckle-tracking echocardiography (2D-STE). *Results:* GLS values presented statistically significant differences between the three groups (*p* < 0.001); markedly increased values (more negative) were observed in the control group (−20.2 ± 1.4%) compared with HTN group values (−18.4 ± 3.0%, *p* = 0.031) and with HFpEF group values (−17.6 ± 2.3%, *p* < 0.001). The correlation between GLS values and HTN stages was significant, direct, and average (Spearman coefficient rho = 0.423, *p* < 0.001). GLS had the greatest ability to detect patients with HFpEF when HFpEF + CAD + HTN diastolic dysfunction (*n* = 30) + CON diastolic dysfunction (*n* = 2) from HFpEF + CAD + HTN + CON was analyzed. (optimal GLS limit of −19.35%, area under curve = 0.833, *p* < 0.001). *Conclusions:* Global longitudinal strain can be used for clinical assessment in differentiating coronary and hypertensive patients at higher risk for development of systolic dysfunction.

## 1. Introduction

Conventional echocardiographic markers of left ventricular (LV) function, despite their widespread use in current diagnostic and management guidelines of heart failure (HF) [1], have significant disadvantages, especially because recently HF with preserved ejection fraction (HFpEF, LVEF ≥50%) became the dominant presentation [2], requiring a reliable assessment of LV function. Therefore, more refined methods are needed. One of these, two-dimensional speckle-tracking echocardiography (2D STE), offers new diagnostic possibilities as a valuable tool for assessing left ventricular function [3]. Thus, global longitudinal strain (GLS) assessment by 2D STE has become a clinically feasible alternative to ejection fraction for quantifying myocardial function. Evidence from studies [4,5,6] has shown a greater sensitivity of GLS to detect early left ventricular systolic function impairment than measuring LVEF and also to provide additional prognostic information. The technology is validated, widely available, and reproducible within an acceptable range [7,8].

A dominant risk factor for HFpEF, regardless of the presence of LV hypertrophy, is hypertension [9], with an incidence of 11–23% reported by the registries of two large studies dedicated to HF [10,11]. The optimal therapy of hypertension in patients with HFpEF (i.e., diastolic dysfunction) is uncertain. The cornerstone of HFpEF management is represented by the management of hypertension, and a personalized matching of antihypertensive treatments to patient phenotype holds great promise for improving outcomes in patients with HFpEF [12]. Guidelines based on LVEF measurement [1] may therefore not include a number of patients who would benefit from early therapy intervention to prevent further myocardial decompensation and future adverse outcomes. The assessment of myocardial strain, or intrinsic deformation, holds promise to improve these issues.

Moreover, GLS has been shown to detect and distinguish the different degrees of subclinical LV dysfunction, constantly improving the risk stratification of patients with heart failure [13]. To provide evidence in support of GLS integration into routine clinical practice, further studies are needed to confirm that such approaches will improve the selection of therapy for these patients and, consequently, the outcomes.

However, it is not well studied how GLS may contribute to the clinical evaluation of HFpEF in hypertensive patients. It is therefore of interest to study this aspect. The aim of the present study was to identify subclinical left ventricular dysfunction among hypertensive subjects compared with subjects with confirmed HFpEF secondary to coronary artery disease vs. control. This study represents a challenge, and it might be helpful in differentiating patients at higher risk for the development of HF.

## 2. Materials and Methods

### 2.1. Study Design and Populations

This retrospective study was conducted between 2019 and 2021 in the Cardiovascular Prevention and Rehabilitation Clinic of the Institute of Cardiovascular Diseases Timisoara, Romania, and included 3 groups of patients, as follows: group 1 (HFpEF) enrolled 62 coronary patients with heart failure with preserved LVEF (>50%) in stable condition, group 2 (HTN) enrolled 46 hypertensive patients, and group 3 (CON) represented the control group consisting of 40 age-matched subjects.

As documented in the individual case history, all patients with coronary heart disease included in the study underwent diagnostic coronary angiography, followed by revascularization procedures. Patients with stable angina were not included in the study. The exclusion criteria also included acute heart failure, congenital heart disease, degenerative valve disease, cardiomyopathies of other causes than CAD, pulmonary, pericardial or rheumatic inflammatory diseases, active infections or known neoplasms, anemia, and renal or hepatic disease (due to similar or identical disease symptoms). Patients with left ventricular ejection fraction <50% were excluded from this study.

All patients with heart failure were staged at the time of hospitalization, according to the New York Heart Association (NYHA) functional classification, and patients with NYHA class IV were excluded. Additionally, based on the presence of typical signs and symptoms, as recommended by the European guidelines in force at the beginning of this study, the diagnosis of heart failure with preserved ejection fraction was made [1]. The patients were selected from 623 hospitalized patients with HF of all causes. Patients with hypertension were staged based on definitions of hypertension grade from the 2018 ESC/ESH Guidelines for the management of arterial hypertension [14]. Patients enrolled in the hypertension group were selected from our data base.

Healthy subjects age-matched to the HFpEF group addressed by primary care physicians to our clinic for cardiovascular disease screening were included in the control group. The CON group included subjects without cardiovascular diseases (coronary heart disease, carotid artery disease, peripheral artery disease, stroke, heart failure), inflammatory diseases, neoplasia, and familial dyslipidemia. Additionally, in this group, systemic blood pressure values and lipid profile parameters were within normal limits without specific treatment, and fasting blood glucose was lower than 100 mg/dL.

The study was approved by the Ethics Commission of the Institute of Cardiovascular Diseases Timisoara (approval certificate 1432/20.02.2019), and the study was conducted in accordance with the principles of the Declaration of Helsinki.

### 2.2. Clinical and Biochemical Evaluation

Clinical and biochemical evaluation was performed in all patients included in the study. Personal data were collected: age, gender, important family history for cardiovascular diseases such as early cardiovascular disease in first degree blood relatives (for subjects aged 55 years or more for males and aged 65 years or more for females), and smoking status. The standard clinical examination included the measurement of systolic blood pressure (SBP) and diastolic blood pressure (DBP) and body mass index (BMI). Blood pressure (BP) was determined with a usual tensiometer (Riester, Germany) with a suitable cuff for the arm of each subject. For each patient, body weight was determined by using a mechanical scale and height was measured with a metal taliometer (Fazzini, Italy). The body mass index was calculated according to the following formula: BMI = weight (kg) ÷ height^2^ (m^2^). Complete blood count, including hemoglobin, white blood cells (WBCs) or leucocytes, were calculated with an automatic hematology analyzer -MINDRAY BC-5300 (Shenzhen, China). The fasting blood glucose was determined by the hexokinase test (HK) using a Siemens Dimension RXL-MAX, Dade Behring device, and reagents. Triglycerides, total cholesterol, and HDL and LDL cholesterol fractions were determined on the same device by photometric methods. For determination of creatinine, the Jaffe method without deproteinization was used. Estimated glomerular filtration rate (eGFR) was calculated based on the MDRD (Modification of Diet in Renal Disease) formula [15]: eGFR = 186 × (Creatinine/88.4)^−1.154^ × (Age)^−0.203^ × (0.742 if female).

### 2.3. Determination of Echocardiographic Parameters and Speckle-Tracking Echocardiography (STE)—Strain Analysis

**Figure 1:**
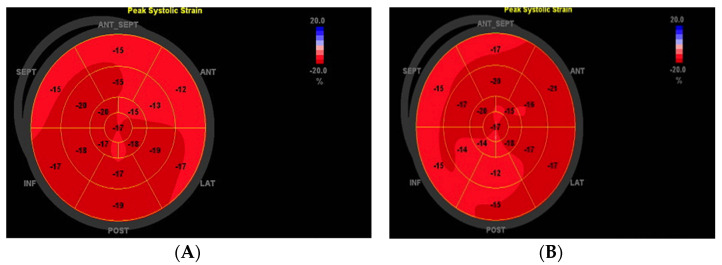
Example illustration of speckle-tracking analysis. (**A**) Assessment of 2-dimensional strain in a patient from HFpEF and (**B**) in a patient from HTN group.

Echocardiography was performed in all patients enrolled in the study on the GE VIVID E9 ultrasound system (manufactured by GEMS Ultrasound, Horten, Norway) equipped with phased-array transducer (M5S). The 2-dimensional (2D) echocardiographic images were acquired from parasternal long- and short-axis views and the three standard apical views. LV wall thickness and chamber size were measured according to the 2005 American Society of Echocardiography (ASE) guideline and standards [16]. Speckle-tracking analysis was performed using dedicated wall motion tracking software: Automated Function Imaging for 2D imaging (from GE Vingmed Ultrasound AS, Horten, Norway). A 17-segment model was used (Figure 1). Values of the peak systolic longitudinal strain from the apical long-axis, apical 4-chamber, and apical 2-chamber views were obtained from automated function imaging software. GLS and longitudinal systolic strain rate were automatically obtained from the three standard apical views. The acquisition for offline analysis was made for 3 consecutive cardiac cycles in each view. The peak longitudinal systolic strain values were measured at basal, mid, and apical regions in six left ventricular standard segments allowing for an accurate segmental analysis, with evaluation of the global longitudinal strain on the resulting bull’s eye model (Figure 1). The investigator visually assessed the detected region of interest (ROI) and, if it was necessary, manually modified the ROI to ensure accurate tracking of the speckles. The patients who had more than 3 inaccurately tracked segments because of low image quality, were excluded from further analysis. The normal GLS is usually in the range of −18% and lower (i.e., more negative) [17]. In Figure 1 are illustrated examples of speckle-tracking analysis from patients enrolled in the study.

### 2.4. Statistical Analysis

Statistical analysis was performed with EpiInfo software (v.7.2.2.6, CDC, Atlanta, GA, USA) and with SPSS software, (version 17, SPSS Inc., Chicago, IL, USA). The data were electronically filed using Microsoft Excel (version 2013, MS Corp., Redmond, WA, USA). For numeric variables, descriptive statistics were performed, and the comparisons between these were made with the non-parametric Kruskal–Wallis test or by determining the Spearman’s correlation coefficient for more than 2 series. The Mann–Whitney test was used for comparisons between two sets of values with no Gaussian distribution. The one-way ANOVA test was used in normal distributed data. Scheffe post hoc analysis was used to adjust significant levels obtained in linear regression analysis to consider multiple comparisons. Performance of global longitudinal strain for the early prediction of impaired systolic function was evaluated by receiver operating characteristics (ROC) analyses. DeLong’s method was used to calculate optimal cutoffs. For nominal variables, frequency tables were elaborated, and the associations between them were evaluated by applying the Chi square(χ2) test. The normal distribution of numerical values was represented by mean ± standard deviation (Shapiro–Wilk test, *p* ≥ 0.05) and the non-normal distribution of numerical values was represented by median (interquartile range)—Shapiro–Wilk test, *p* < 0.05. The results were considered significant for a value of *p* < 0.05.

## 3. Results

### 3.1. Patient Population and Characteristics

Table 1 contains, described in detail, the clinical, biochemical, and demographic features of the patients.

Patients with HFpEF were older compared with HTN and CON groups (62.9 ± 8.57 years vs. 61 ± 11.72 years vs. 60.2 ± 8.73 years, *p* = 0.385), but no statistically significant difference was observed between groups. They also tended to have a higher BMI compared with the other groups (29.9 ± 4.47 kg/m^2^ for HFpEF vs. 28.4 ± 4.31 kg/m^2^ for HTN and 25.5 ± 2.08 kg/m^2^ for CON, *p* < 0.001). Regarding gender, no difference between groups was observed (67.7% for HFpEF vs. 52.2% for HTN vs. 55.0% for CON, *p* = 0.231). Although no statistically significant differences were observed between groups in terms of diastolic blood pressure (*p* = 0.458) and heart rate (*p* = 0.768), in the HFpEF group, all patients (*n* = 62) had hypertension, and 17.4% (*n* = 8) had atrial fibrillation previously.

In addition, a statistically significant difference was observed between groups regarding the presence of chronic kidney disease (considered for a value of eGFR < 60 mL/kg/1.73 m^2^; i.e., 9.7% for HFpEF vs. 21.75% for HTN vs. 0.0% for CON, *p* = 0.032) and type 2 diabetes mellitus (24.2% HFpEF vs. 34.8% for HTN vs. 0.0% for CON). All patients from the HFpEF group were staged at the time of hospitalization (at admission and at discharge) according to the New York Heart Association functional classification. On admission, 9 patients (14.5%) had NYHA class 0, 37 patients (59.7%) had NYHA class II, and 16 patients (25.8%) had NYHA class III. Upon discharge, only 1 patient (1.6%) remained classified as NYHA class III, while 24 patients (38.7%) had NYHA class II, and the same number of patients had NYHA class I. Additionally, at discharge, 13 patients (21.0%) had NYHA class 0. Hypertension was absent in the CON group. Stage 1 HTN was present in 3 patients (6.5%) from HTN group and 10 patients (16.1%) from HFpEF, stage 2 HTN in 28 patients (60.9%) from HTN group and 22 patients (35.5%) from HFpEF group, and stage 3 HTN in 15 patients (32.6%) from HTN group and 30 patients (48.4%) from HFpEF group, with statistically significant differences between the groups (*p* < 0.001). No statistically significant differences were found between the three groups for hemoglobin values (*p* = 0.339), white blood cells (*p* = 0.186), fasting blood glucose (*p* = 0.159), potassium (*p* = 0.939), and sodium (*p* = 0.300). A statistically significant difference was observed for creatinine values between groups (1.0 ± 0.23 mg/dL vs. 1.1 ± 0.41 mg/dL vs. 0.9 ± 0.14 mg/dL, *p* = 0.011), related to the presence of chronic kidney disease. (Table 1) Scheffe post hoc analysis was used to adjust significance.

Analyzing the cardiovascular risk factors, we observed that only the triglyceride values did not present statistically significant differences between the three groups (*p* = 0.249) (Table 1).

The comparison of prevalent use of medications in the three groups (*n* = 148) is reported in Table 2. In the HFpEF group, 46.8% (*n* = 29) used ACEi or ARB compared with 54.34% (*n* = 25) in the HTN group and 0% in the CON group (*p* = 0.043); 66.1% (*n* = 41) used beta-blockers compared with 39.2% (*n* = 18) in the HTN group and with 2.5% (*n* = 1) in the CON group (*p* < 0.001); and 17.7% (*n* = 11) used calcium channel blockers compared with 19.56% (*n* = 9) in the HTN group and 0% in the CON group (*p* = 0.002). Statistically significant differences were found between the three groups regarding the use of amiodarone (*p* = 0.019), aldosterone antagonist (*p* = 0.001), furosemide (*p* = 0.002), aspirin (*p* < 0.001), antiplatelet agent (*p* < 0.001), and anticoagulant (*p* = 0.035). In the HFpEF group, 87.1% (*n* = 54) used statin compared with 80.4% (*n* = 37) in the HTN group and with 0% in the CON group (*p* < 0.001). Moreover, 12.9% (*n* = 8) in the HFpEF group and 10.9% (*n* = 5) in the HTN group used fibrates (*p* = 0.011) as anti-dyslipidemic drugs. A total of 15 patients from HFpEF used oral antidiabetic agents (24.2%), and 3 patients used insulin, while in the HTN group 15 patients used oral antidiabetic agents (32.60%) and only 1 patient used insulin.

In Table 3 the type of cardiovascular revascularization procedures and the coronary arteries involved in the HFpEF group are described. From the CAD patients, 20 (32.25%) received coronary artery bypass grafting, 51.61% (*n* = 32) underwent percutaneous transluminal coronary angioplasty, and 10 patients (16.15%) received thrombolytic therapy. The coronary arteries involved were the following: right coronary artery in 30 patients (48.38%), left anterior descending artery in 23 patients (37.1%), and circumflex artery and its branches in 20 patients (32.3%).

Echocardiographic data for all patients (*n* = 148) are summarized in Table 4. No statistically significantly differences were found regarding LVEF (%) between the three groups (55.0 ± 5.0% for HFpEF group vs. 56.0 ± 8% for HTN group vs. 56.0 ± 4.0% for CON group, *p* = 0.262). Values of IVSd (cm) and LVPWd (cm) were not statistically significant when compared with HTN and HFpEF groups. LVEDV (mL) values were significantly lower in the CON group compared with HTN group (*p* < 0.001) and with HFpEF group (*p* = 0.004) and in the HFpEF group compared with HTN group (*p* = 0.002). LVESV (mL) values were significantly lower in the CON group compared with HTN group and with HFpEF group (*p* < 0.001). E-mitral wave values were significantly lower in the HTN group compared with CON group (*p* = 0.029) and with HFpEF group (*p* = 0.001), and also in the CON group compared with HFpEF group (*p* = 0.042). Regarding A-mitral wave values, significantly lower values were observed in the CON group vs. HFpEF (*p* = 0.002) and vs. HTN group (*p* < 0.001) and also when HFpEF and HTN groups (*p* = 0.007) were compared. In the HTN group, 30 patients had diastolic dysfunction (E/A < 1), while 36 had LVH. In the CON group two patients had diastolic dysfunction, probably due to age.

### 3.2. Speckle-Tracking Echocardiographic Parameters

**Figure 2:**
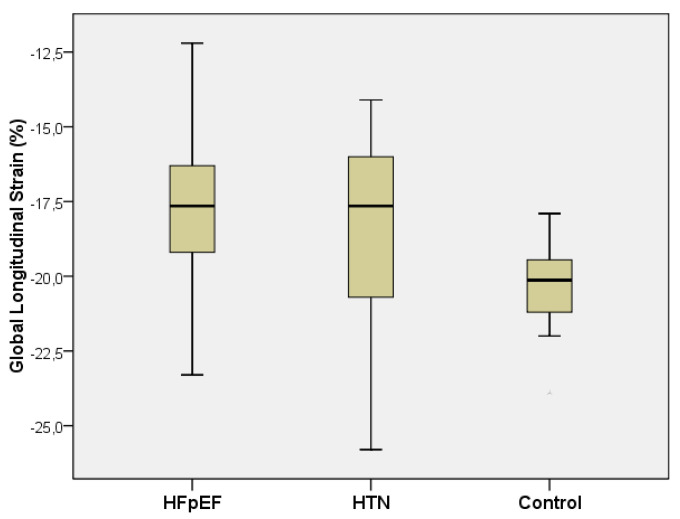
Boxplot representing GLS values compared between the three groups (*n* = 148).

There were significant differences between GLS (%) values between the three groups (one-way ANOVA test, *p* < 0.001); marked increased values (more negative) were observed in the control group (−20.2 ± 1.4) compared with the HFpEF group (−17.6 ± 2.3) (Scheffe post hoc test, *p* < 0.001) and with the HTN group (−18.4 ± 3.0), *p* = 0.031) (Figure 2).

**Figure 3:**
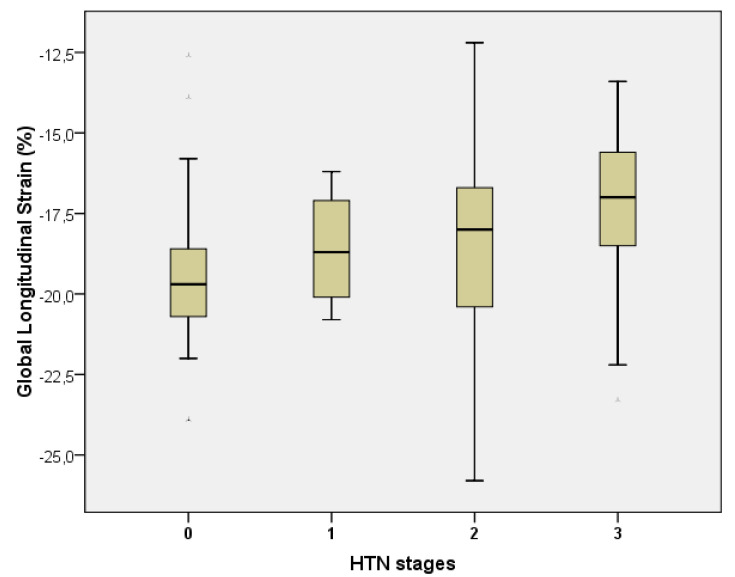
Boxplot for GLS values, comparing HTN stages in all patients vs. CON (*n* = 148).

By applying the one-way ANOVA test, it was observed that GLS (%) values decreased (were more positive) as arterial hypertension stages increased (*p* = 0.012). GLS values were significantly reduced in patients with hypertension stage 3 compared with normotensive patients (*p* = 0.020), hypertension stage 1 (*p* = 0.035), and stage 2 (*p* = 0.022) (Scheffe post hoc test). The correlation between GLS values and HTN stages for the entire group was significant, direct, and average (Spearman coefficient rho = 0.423, *p* < 0.001) (Figure 3).

**Figure 4:**
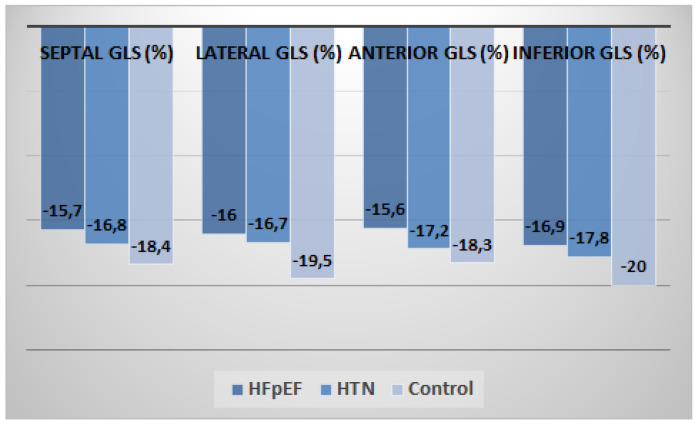
Distribution of mean regional longitudinal strain values at basal level between the three groups (*n* = 148).

In 2D speckle-tracking echocardiography, the HFpEF group showed a marked reduction in segmental LS compared with the hypertension group or age-matched control group at basal level (*p* = 0.017 for septal region, *p* = 0.025 for lateral region, *p* = 0.020 for anterior region and *p* = 0.019 for inferior region) (Figure 4).

**Figure 5:**
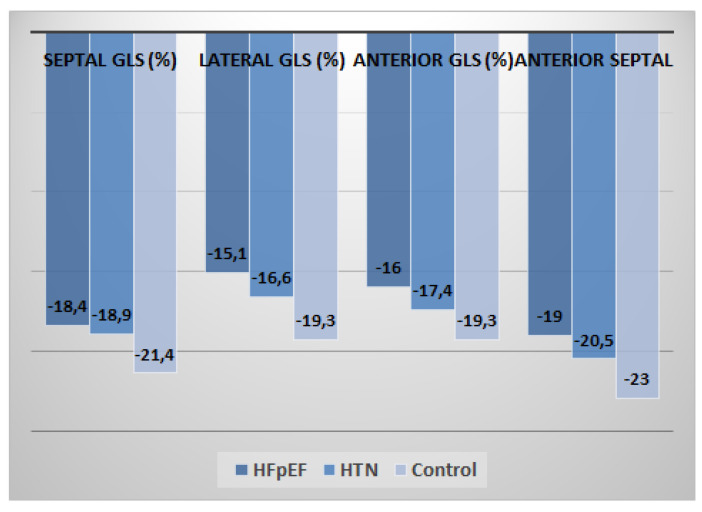
Distribution of mean regional longitudinal strain values at mid-level between the three groups (*n* = 148).

At mid-level, a reduction in LS values was found in the HFpEF group compared with the HTN group and age-matched control group (*p* = 0.029 for septal region, *p* = 0.004 for lateral region, *p* = 0.022 for anterior region and *p* = 0.011 for inferior region) (Figure 5).

**Figure 6:**
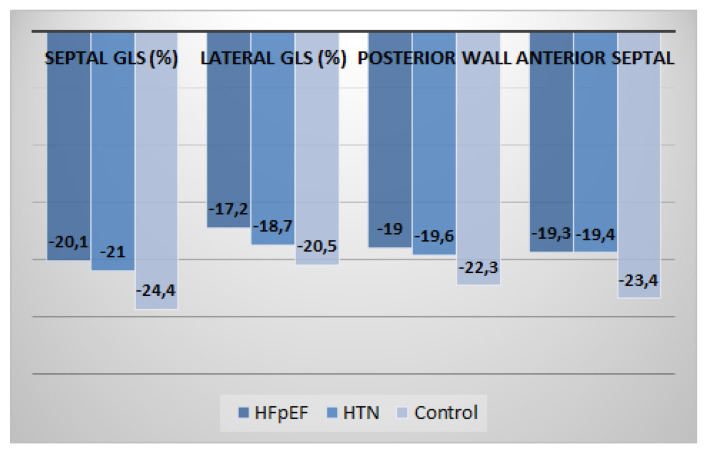
Distribution of mean regional LS values at apical level between the three groups (*n* = 148).

At apical level, the HFpEF group showed marked reduction in LS compared with HTN and CON groups (*p* = 0.004 for septal region, *p* = 0.034 for lateral region, *p* = 0.029 for anterior region and *p* = 0.016 for inferior region) (Figure 6).

**Figure 7:**
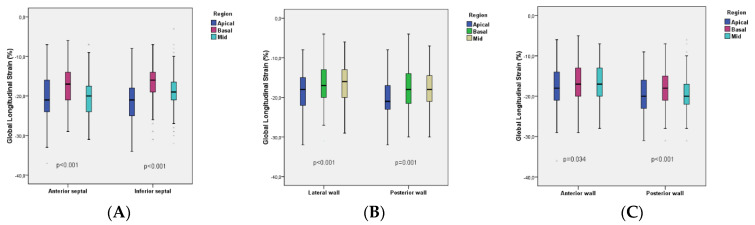
The range of peak longitudinal systolic strain values at basal, mid, and apical regions in the six left ventricular standard segments. The best values were obtained in basal regions at anterior and inferior septal levels (*p* < 0.001, in both, **A**), mid regions at lateral wall (*p* < 0.001) and posterior wall levels (*p* = 0.001) (**B**), and basal regions at anterior wall (*p* = 0.034) and posterior wall levels (*p* < 0.001) (**C**).

Peak longitudinal systolic strain values were compared at basal, mid, and apical regions of anterior and inferior septal levels, lateral and posterior wall levels, and anterior and posterior wall levels. The best values were obtained in basal regions at anterior and inferior septal levels (*p* < 0.001, in both, Figure 7A), mid regions at lateral wall (*p* < 0.001) and posterior wall levels (*p* = 0.001) (Figure 7B), and basal regions at anterior wall (*p* = 0.034) and posterior wall levels (*p* < 0.001) (Figure 7C).

### 3.3. Diagnostic Performance of Global Longitudinal Strain

**Figure 8:**
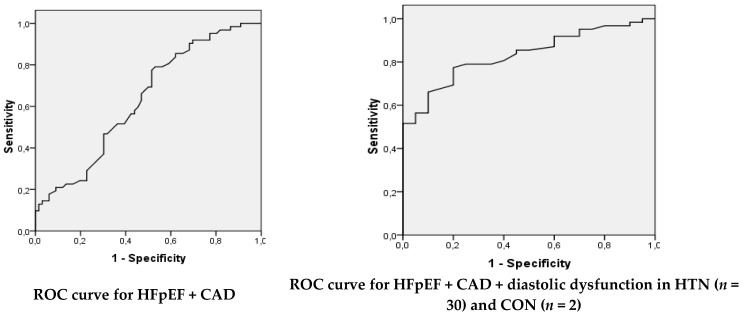
ROC curves for predictive performance of GLS for HFpEF.

To analyze the predictive performance of global longitudinal strain for the diagnosis of HFpEF, a ROC curve analysis was performed (Table 5, Figure 8). We determined the predictive value of GLS in HFpEF + CAD patients from HFpEF + CAD + HTN + CON and also in HFpEF + CAD + HTN with diastolic dysfunction (*n* = 30) + CON with diastolic dysfunction (*n* = 2) from HFpEF + CAD + HTN + CON. When HFpEF + CAD was analyzed, the performance of GLS in predicting HFpEF was satisfactory. The optimal cutoff of GLS in this case was −19.45%, with sensitivity = 79.03%, specificity = 46.97%, PPV = 58.33%, NPV = 70.45%. An optimal cutoff of GLS of −19.35 (%), with sensitivity = 77.42%, specificity = 80.00%, PPV = 92.31%, NPV = 53.33%, an AUC = 0.833, *p* < 0.001, and a very good performance of GLS in predicting HFpEF was also obtained when HFpEF + CAD + HTN with diastolic dysfunction (*n* = 30) + CON with diastolic dysfunction (*n* = 2) from HFpEF + CAD + HTN + CON was analyzed.

## 4. Discussion

In this study, we examined subclinical left ventricular dysfunction among hypertensive subjects compared with subjects with HFpEF secondary to coronary artery disease vs. control and associations of LV GLS with other baseline characteristics in these patients. This study has three major findings. Our main finding was reduced GLS values with significant differences between the three groups. Second, longitudinal strain was reduced in all regions. Third, the diagnostic performance of GLS in predicting HFpEF was good, with the best cutoff value of −19.35 (%).

Our results are based on other studies analyzing LV GLS in patients with HFpEF. They are similar to the findings of Kim et al., who also observed that GLS is reduced in HFpEF patients compared with hypertensive patients and normal subjects in decreasing order (−19.88 ± 2.04% vs. −17.75 ± 3.12 vs. −15.52 ± 5.32, (*p* < 0.001) [18]. In our analysis, we found a similar relationship between the three groups. Investigators from PARAMOUNT (Prospective Comparison of ARNI with ARB on Management of Heart Failure with Preserved Ejection Fraction Trial) also showed that HFpEF patients demonstrated significantly lower GLS values when comparing controls and hypertensive patients (−20.0 ± 2.1 vs. −14.6 ± 3.3, *p* < 0.0001) [19].

In our study, no statistically significant differences were found regarding LVEF (%) between the three groups. Thus, GLS was confirmed to be a more refined measurement for detecting left ventricular systolic dysfunction. In the HTN group, GLS values were reduced (more positive) the more the arterial hypertension stages increased (*p* = 0.012). The correlation between GLS values and HTN stages was significant, direct, and average (Spearman coefficient rho = 0.423, *p* < 0.001). In a previous study performed on 200 hypertensive patients, GLS ranged from −25% to −11.6% (mean −16.9 ± 3.2%). The univariate analysis showed an association between reduced GLS and hypertension lasting for over 10 years (odds ratio (OR) = 3.51, 95% confidence interval (CI) 1.73–7.09; *p* = 0.002), a correlation also valid for uncontrolled hypertension (OR = 3.55, 95% CI 1.96–6.43; *p* < 0.0001) [20]. Statistically lower GLS values were also found in a study that compared 38 newly diagnosed, never-treated hypertensives with 38 control healthy subjects (−18.3 ± 2.1% vs. −20.9 ± 2.7%, *p* < 0.0001). LVEF values were not significantly different between the two groups (55.3 ± 4.4% vs. 56.3 ± 5.8%, *p* = NS) [21]. Thus, the results of this study, similar to those of previous studies, emphasize the need to perform a comprehensive cardiac evaluation in hypertensive patients other than the simple chamber function currently expressed by LVEF, which should also include GLS assessment. In particular, this is recommended because 2D STE has been shown to detect substantial impairments of LV systolic function in hypertensive patients with preserved LVEF, which has led to identification of subgroups at higher risk selected for subsequent more aggressive preventive measures [22].

The role of GLS in the prediction of left ventricular filling pressure in patients with coronary artery disease and normal LVEF was analyzed in a study on 84 patients with CAD and 30 healthy controls. Compared with controls, the patients with CAD had significantly lower GLS [23]. In patients with stable ischemic heart disease who underwent strain echocardiography and coronary angiography, the cutoff value of GLS to detect significant CAD was −16.5 (%) (87.6% sensitivity, 85.7% specificity, *p* < 0.0001), and the agreement between GLS and coronary angiography for detection of significant CAD was substantial (*κ* = 0.676, *p* < 0.0001). The investigators concluded that GLS must be assessed in subjects with stable ischemic heart disease to rule out significant CAD even if conventional echocardiographic parameters are normal [23]. In our study, an optimal cutoff of GLS of −19.35 (%) and a very good performance of GLS in predicting HFpEF was obtained when the sample HFpEF + CAD + diastolic dysf in HTN (*n* = 30) and CON (*n* = 2) was analyzed. Prior to our study, GLS proved to be a powerful diagnostic tool for assessing the risk of stable coronary heart disease in people with normal LVEF [24].

Moreover, there are several strengths of the current study. According to previous studies that investigated GLS values, strain values are reduced with increasing age [25,26]. It was shown in a study that included 266 consecutive healthy subjects without cardiovascular risk factors, with a mean age of 39.2 ± 17.5 years, where 137 of participants were female, that GLS (*p* < 0.001) values were progressively reduced with increasing age, and post hoc intra-group analysis showed that the decline in GLS was significant in the decades 50–60 and ≥60 [25]. Compared with other studies with similar samples that had significant differences in age between groups [18,27], in our study the groups were similar in terms of age, so we believe that our strict inclusion criteria and data collection have minimized the bias.

Furthermore, in the current study, we provide longitudinal strain values for representative segments of the LV (Figure 4, Figure 5 and Figure 6). Peak longitudinal systolic strain values were also compared at basal, mid, and apical regions of anterior and inferior septal levels, lateral and posterior wall levels, and anterior and posterior wall levels. The best values were obtained in basal regions at anterior and inferior septal levels, mid regions at lateral wall and posterior wall levels, and basal regions at anterior wall and posterior wall levels. This could be another added value of the current study. We observed that peak systolic segmental, regional (basal, mid, and apical), and GLS are reduced in patients with HFpEF vs. HTN patients vs. control age-matched healthy subjects.

At the same time, our study demonstrates that although LVEF will remain a cornerstone of LV function assessment, the addition of GLS for clinical assessment allows for more detailed phenotyping and improved risk assessment, making it a tool for present and future therapeutic progress.

### Study Limitations

Our study has several limitations, including the fact that it was a retrospective study from a clinical database and patients met specific inclusion criteria—for example, the diagnosis of coronary heart disease and HFpEF in stable condition. At the same time, all data were extracted from patient records, which limits the ability to objectively assess the relationship between GLS and impaired functional capacity, an important hallmark of HFpEF syndrome. Patients included in this study may not be representative of HFpEF patients in the community due to specific inclusion and exclusion criteria in the study.

## 5. Conclusions

This study demonstrated that global and regional longitudinal strain can detect myocardial dysfunction early and may be an appropriate target for preventive strategies, as it occurs before LVEF abnormalities. The results of this study sustain the contribution of GLS to clinical assessment of HFpEF in coronary and hypertensive patients. In addition, from the perspective of early identification to monitoring left ventricular systolic dysfunction, it is very necessary to promote GLS evaluation in routine clinical practice in all these patients.

## Figures and Tables

**Table 1:** Characteristics of all patients (*n* = 148).

Variables	HFpEF	HTN	Control	P ^test^
***n***	**62**	**46**	**40**	
**Age, years**	62.9 ± 8.57	61 ± 11.72	60.2 ± 8.73	0.385 ^b^
**Male sex, n (%)**	42 (67.7%)	24 (52.2%)	11 (55.0%)	0.231 ^b^
**SBP, mmHg**	122.4 ± 21.61	136.5 ± 18.19	125.3 ± 9.89	0.018 ^a^*
**DBP, mmHg**	81.5 ± 12.91	79 ± 11.86	78.6 ± 9.16	0.458 ^a^
**HR, bpm**	72.5 ± 19.94	71 ± 12.56	74.1 ± 10.75	0.768 ^a^
**AF, n (%)**	8 (17.4%)	3 (4.8%)	0 (0.0%)	0.010 ^b^*
**CKD ^#^ (yes)**	6 (9.7%)	10 (21.7%)	0 (0.0%)	0.032 ^b^*
**NYHA classification at admission**				
**0**	9 (14.5%)	46 (100.0%)	40 (100.0%)	<0.001 ^b^*
**II**	37 (59.7%)	0 (0.0%)	0 (0.0%)	
**III**	16 (25.8%)	0 (0.0%)	0 (0.0%)	
**NYHA classification at discharge**				
**0**	13 (21.0%)	46 (100.0%)	40 (100.0%)	<0.001 ^b^*
**I**	24 (38.7%)	0 (0.0%)	0 (0.0%)	
**II**	24 (38.7%)	0 (0.0%)	0 (0.0%)	
**III**	1 (1.6%)	0 (0.0%)	0 (0.0%)	
**HTN stages**				
**0**	0 (0%)	0 (0.0%)	40 (100.0%)	<0.001 ^b^*
**1**	10 (16.1%)	3 (6.5%)	0 (0.0%)	
**2**	22 (35.5%)	28 (60.9%)	0 (0.0%)	
**3**	30 (48.4%)	15 (32.6%)	0 (0.0%)	
**Hemoglobin (g/dL)**	14.1 ± 1.64	14 ± 1.45	13.5 ± 1.46	0.339 ^a^
**WBCs (×10^9^/L)**	7.8 ± 2.59	7.0 ± 1.58	7.7 ± 1.64	0.186 ^a^
**Fasting blood glucose (mg/dL)**	117.5 ± 40.08	116.6 ± 43.25	99.2 ± 6.26	0.159 ^a^
**Creatinine (mg/dL)**	1.0 ± 0.23	1.1 ± 0.41	0.9 ± 0.14	0.011 ^a^*
**K^+^ (mmEq/L)**	4.2 ± 0.42	4.2 ± 0.39	4.2 ± 0.24	0.939 ^a^
**Na^+^ (mmEq/L)**	140.5 ± 5.83	140.8 ± 4.17	142.4 ± 2.23	0.300 ^a^
**Risk factors**				
**Hypertension (yes)**	53 (85.48%)	46 (100%)	0 (0%)	<0.001 ^b^*
**Smoking (yes)**	17 (27.41%)	15 (32.60%)	9 (22.5%)	<0.001 ^b^*
**BMI, kg/m^2^**	29.9 ± 4.47	28.4 ± 4.31	25.5 ± 2.08	<0.001 ^a^*
**TC, mg/dL**	174.5 ± 31.45	181.8 ± 34.21	165 ± 19.75	0.021 ^a^*
**LDL-c, mg/dL**	109.8 ± 44.78	120.7 ± 53.13	88.9 ± 17.12	0.034 ^a^*
**HDL-c, mg/dL**	42.13 ± 8.55	39.46 ± 9.31	51.38 ± 9.66	<0.001 ^a^*
**TG, mg/dL**	142.4 ± 65.45	144.0 ± 85.37	114.9 ± 23.72	0.249 ^a^
**T2DM, n (%)**	15 (24.2%)	16 (34.8%)	0 (0.0%)	0.010 ^b^*

SBP, systolic blood pressure; DBP, diastolic blood pressure; HR, heart rate; AF, atrial fibrillation; CKD, chronic kidney disease, K^+^, potassium; Na^+^, sodium; T2DM, type 2 diabetes mellitus; NYHA, the New York Heart Association functional classification; WBCs, white blood cells; BMI, body mass index; TC, total cholesterol; HDL-c, high-density lipoprotein cholesterol; LDL-c, low-density lipoprotein cholesterol, TG, triglycerides. ^#^ for a value of eGFR < 60 mL/kg/1.73 m^2^. Values are expressed as mean ± standard deviation (SD). ^test^—used statistical test; ^a^—one-way ANOVA in normal distributed data; ^b^—Kruskal–Wallis in non-normal distributed data; *—significant difference.

**Table 2:** Comparison of prevalent use of medications in the three groups (*n* = 148).

Variables	HFpEF	HTN	Control	P ^test^
***n***	**62**	**46**	**40**	
**Medications**				
**ACEi or ARB**	29 (46.8%)	25 (54.34%)	0 (0.0%)	0.043 ^b^*
**Beta-Blocker**	41 (66.1%)	18 (39.2%)	1 (2.5%)	<0.001 ^b^*
**Amiodarone**	7 (11.3%)	1 (2.17%)	0 (0.0%)	0.019 ^b^*
**Aldosterone Antagonists**	22 (35.5%)	12 (26.1%)	0 (0.0%)	0.001 ^b^*
**Furosemide**	21 (33.9%)	5 (10.9%)	0 (0.0%)	0.002 ^b^*
**Aspirin**	48 (77.4%)	23 (50.0%)	0 (0.0%)	<0.001 ^b^*
**Antiplatelet Agent**	40 (64.5%)	19 (41.3%)	0 (0.0%)	<0.001 ^b^*
**Anticoagulant**	6 (9.7%)	5 (10.9%)	0 (0.0%)	0.035 ^b^*
**Statin**	54 (87.1%)	37 (80.4%)	0 (0.0%)	<0.001 ^b^*
**Calcium Channel Blocker**	11 (17.7%)	9 (19.56%)	0 (0.0%)	0.002 ^b^*
**Fibrates**	8 (12.9%)	5 (10.9%)	0 (0.0%)	0.011 ^b^*
**Oral antidiabetic agents (metformin, sitagliptin)**	15 (24.2%)	15 (32.60%)	0	<0.001 ^b^*
**Insulin**	3 (4.83%)	1 (2.17)	0	0.005 ^b^*

ACEi or ARB, angiotensin converting enzyme inhibitors or angiotensin receptor blockers. Values are expressed as mean ± standard deviation (SD). ^test^—used statistical test; ^b^—Kruskal–Wallis in non-normal distributed data; *—significant difference.

**Table 3:** Type of cardiovascular revascularization procedures and the coronary arteries involved (*n* = 148).

Variables	HFpEF
***n***	**62**
**The type of cardiovascular revascularization procedures**	
**CABG (yes)**	20 (32.25%)
**PTCA (yes)**	32 (51.61%)
**Thrombolyzed myocardial infarction (yes)**	10 (16.1%)
**The coronary arteries involved**	
**RCA (yes)**	30 (48.38%)
**LAD (yes)**	23 (37.1%)
**Cx (yes)**	20 (32.3%)

CABG, coronary artery bypass grafting; PTCA, percutaneous transluminal coronary angioplasty; RCA, right coronary artery; LAD, left anterior descending artery; Cx, circumflex artery and its branches.

**Table 4:** Echocardiographic data for all patients (*n* = 148).

Variables	HFpEF	HTN	Control	P ^test^
***n***	**62**	**46**	**40**	
**Echocardiographic parameter**				
**IVSd (cm)**	1.1 ± 0.2	1.1 ± 0.2	1.0 ± 0.1	0.252 ^b^*
**LVPWd (cm)**	1.1 ± 0.1	1.1 ± 0.2	1.0 ± 0.1	0.273 ^b^*
**LVEDD (cm)**	4.7 ± 0.4	4.5 ± 0.5	4.5 ± 0.2	**0.103 ^a^**
**LVEDV (mL)**	90.0 ± 11.5	100.0 ± 33.5	86.0 ± 10.5	<0.001 ^b^*
**LVESV (mL)**	39.5 ± 9	40.0 ± 6.5	32.0 ± 2.8	<0.001 ^b^*
**LAVI by area-length method (mL/m^2^)**	37.7 ± 6.7	37.5 ± 5.5	35.3 ± 5.6	**0.293 ^a^**
**LVEF (%)**	55.0 ± 5.0	56.0 ± 8	56.0 ± 4.0	**0.262 ^b^**
**E (m/s)**	0.8 ± 0.2	0.6 ± 0.4	0.8 ± 0.1	0.002 ^b^*
**A (m/s)**	0.6 ± 0.1	0.7 ± 0.2	0.5 ± 0.1	<0.001 ^a^*
**E/A < 1 (yes)**	62 (100%)	30 (65.21%)	2 (5%)	<0.001 ^b^*
**LVH (yes)**	62 (100%)	36 (78.26%)	0 (0%)	0.001 ^b^*

IVSd, interventricular septum thickness at end-diastole; LAVI, left atrial volume index; LVH, LVPWd, left ventricular posterior wall thickness at end-diastole; LVEDD, left ventricular nd-diastole dimension; LVEDV, left ventricular end-diastolic volume; LVESV, left ventricular end-sistolic volume; LCEF, left ventricular ejection fraction; LVH, left ventricular hypertrophy, ^test^—used statistical test; ^a^—one-way ANOVA in normal distributed data; ^b^—Kruskal–Wallis in non-normal distributed data; *—significant difference.

**Table 5:** Predictive performance of global longitudinal strain (%) for the diagnosis of HFpEF.

Sample	Cutoff	AUC	Std. Error	Asymptotic Sig.	Asymptotic 95% Confidence Interval		SN (%)	SP (%)	PPV (%)	NPV (%)
					Lower Bound	Upper Bound				
HFpEF + CAD	−19.45	0.632	0.049	0.010 *	0.536	0.728	79.03	46.97	58.33	70.45
HFpEF + CAD + diastolic dysf in HTN (*n* = 30) and CON (*n* = 2)	−19.35	0.833	0.045	<0.001 *	0.746	0.921	77.42	80.00	92.31	53.33

*—significance; AUC—area under the curve; SN—sensitivity; SP—specificity; PPV—positive predictive value; NPV—negative predictive value.
